# Adaptive neuroplasticity in the default mode network contributing to absence of central sensitization in primary dysmenorrhea

**DOI:** 10.3389/fnins.2023.1094988

**Published:** 2023-02-09

**Authors:** Lin-Chien Lee, Ya-Yun Chen, Wei-Chi Li, Ching-Ju Yang, Ching-Hsiung Liu, Intan Low, Hsiang-Tai Chao, Li-Fen Chen, Jen-Chuen Hsieh

**Affiliations:** ^1^Institute of Brain Science, College of Medicine, National Yang Ming Chiao Tung University, Taipei, Taiwan; ^2^Integrated Brain Research Unit, Division of Clinical Research, Department of Medical Research, Taipei Veterans General Hospital, Taipei, Taiwan; ^3^Department of Physical Medicine and Rehabilitation, Cheng Hsin General Hospital, Taipei, Taiwan; ^4^Department of Biological Science and Technology, College of Biological Science and Technology, National Yang Ming Chiao Tung University, Hsinchu, Taiwan; ^5^Institute of Traditional Medicine, College of Medicine, National Yang Ming Chiao Tung University, Taipei, Taiwan; ^6^Department of Neurology, Lotung Poh-Ai Hospital, Yilan, Taiwan; ^7^Department of Obstetrics and Gynecology, Taipei Veterans General Hospital, Taipei, Taiwan; ^8^Institute of Biomedical Informatics, National Yang Ming Chiao Tung University, Taipei, Taiwan; ^9^Brain Research Center, National Yang Ming Chiao Tung University, Taipei, Taiwan; ^10^Center for Intelligent Drug Systems and Smart Bio-Devices, National Yang Ming Chiao Tung University, Hsinchu, Taiwan

**Keywords:** primary dysmenorrhea, menstrual pain, central sensitization, pain hypersensitivity, default mode network, neuroplasticity, noxious heat, functional magnetic resonance imaging

## Abstract

**Introduction:**

Primary dysmenorrhea (PDM), the most prevalent gynecological problem among women of reproductive age, presents as a regular pattern of cyclic menstrual pain. The presence or absence of central sensitization (i.e., pain hypersensitivity) in cases of PDM is a contentious issue. Among Caucasians, the presence of dysmenorrhea is associated with pain hypersensitivity throughout the menstrual cycle, indicating pain amplification mediated by the central nervous system. We previously reported on the absence of central sensitization to thermal pain among Asian PDM females. In this study, functional magnetic resonance imaging was used to reveal mechanisms underlying pain processing with the aim of explaining the absence of central sensitization in this population.

**Methods:**

Brain responses to noxious heat applied to the left inner forearm of 31 Asian PDM females and 32 controls during their menstrual and periovulatory phases were analyzed.

**Results and discussion:**

Among PDM females experiencing acute menstrual pain, we observed a blunted evoked response and de-coupling of the default mode network from the noxious heat stimulus. The fact that a similar response was not observed in the non-painful periovulatory phase indicates an adaptive mechanism aimed at reducing the impact of menstrual pain on the brain with an inhibitory effect on central sensitization. Here we propose that adaptive pain responses in the default mode network may contribute to the absence of central sensitization among Asian PDM females. Variations in clinical manifestations among different PDM populations can be attributed to differences in central pain processing.

## Introduction

Affecting more than half of menstruating women worldwide, primary dysmenorrhea (PDM) refers to menstruation-related pain that is not associated with identifiable organic causes (Berkley, 2013). PDM subjects suffer from cramping pain cyclically emanating from the lower abdomen, beginning with the onset of menstrual flow and lasting 24–72 h (i.e., days 1–3 of each menstrual cycle). PDM also manifests as anxious and depressive symptoms and low self-rated quality of life (Lee et al., 2014). Researchers have proposed early-onset PDM as a plausible clinical precipitant for many chronic pain disorders that develop later in life, including irritable bowel syndrome, interstitial cystitis/painful bladder syndrome, chronic low back pain, chronic headache, and fibromyalgia (Berkley, 2013).

Recent neuroimaging studies have revealed evidence suggesting that the brains of PDM females undergo reorganization (adaptive and maladaptive) in response to long-term dysmenorrhea. It is likely that maladaptive functional and structural alterations in the brain underpin the pathophysiology of PDM and corresponding sensory and affective elements of pain. They may also contribute to susceptibility to chronic pain disorders in later life (Tu et al., 2009, 2010; Wei et al., 2016a). In a previous study on the interaction of large-scale resting-state brain networks, we determined that among PDM females, the adaptive role of the default mode network (DMN) involves a dynamic transition from affective processing of pain salience to cognitive modulation of pain (Wu et al., 2016). In the context of interactions among large-scale brain networks, it appears that adaptive neuroplasticity may help to preserve the integrity of functional brain architecture, indicating a lack of overt psychosocial disturbances (Lee et al., 2018).

Central sensitization is defined as enhanced nociceptive signaling within the central nervous system causing hypersensitivity to pain (Woolf, 2011; Farmer et al., 2012). It can cause allodynia or hyperalgesia outside the referred area of pain (i.e., in remote or asymptomatic body sites) indicating pain amplification mediated by the central nervous system. The presence or absence of central sensitization in PDM is a contentious issue. Among Caucasians, the presence of dysmenorrhea is associated with elevated sensitivity to pain (regardless of location) throughout the menstrual cycle (Iacovides et al., 2015; Payne et al., 2017). Inconsistencies in the findings related to pain sensitivity among dysmenorrheic females can be partly explained by differences in experimental methodologies, such as the choice of noxious stimuli (thermal, electric, pressure, or ischemic pain), the location/tissue and the depth of experimental pain stimulation (somatic or visceral tissues), and outcome measures (thresholds or tolerance of pain) (Iacovides et al., 2015; Payne et al., 2017). In a previous study, we detected no evidence of central sensitization to cutaneous thermal pain among young Asian PDM females (Lee et al., 2014). This suggests that pain sensitivity may vary as a function of ethnic or psychosocial characteristics (Cardenas et al., 2004; Rahim-Williams et al., 2012). In the current study, we posit that variations in clinical manifestations among different PDM populations could be attributed to central processing and pain modulation in response to cyclic menstrual pain.

Pain is a complex, multidimensional experience with nociceptive, affective, and cognitive dimensions (Peyron et al., 2000; Garcia-Larrea and Peyron, 2013). Human neuroimaging studies have revealed a constellation of brain regions that are activated during the pain response, including the insula, the primary and secondary somatosensory cortices, the prefrontal cortex, thalamus, anterior and posterior cingulate, basal ganglia, and cerebellum (Coghill et al., 1999; Peyron et al., 2000; Hofbauer et al., 2001; Apkarian et al., 2005; Tracey, 2005; Schweinhardt and Bushnell, 2010). Note that these brain regions comprise the pain network. The tryptophan–kynurenine pathway and its metabolites also play an important role in neuroinflammation, which is intricately linked to the pathogenesis of chronic pain disorders. The tryptophan–kynurenine pathway provides a window by which to investigate the contribution of psychosocial and behavioral factors to central sensitization and the subsequent development of chronic pain disorders (Tanaka et al., 2021).

The mechanisms underlying central pain processing can be investigated by examining the brain responses evoked by inducing pain in an experimental setting (e.g., noxious thermal, electrical, pressure, or ischemic stimuli) while performing functional magnetic resonance imaging (fMRI). Neuroimaging studies have revealed that in several pain-related regions of the brain (e.g., the prefrontal cortex), the central processing of acute pain by patients with chronic pain disorders differs from that of healthy subjects (Apkarian et al., 2005; Schweinhardt and Bushnell, 2010). The prefrontal cortex is involved in the affective, cognitive, interoceptive, and memory components of the pain experience (Apkarian et al., 2005; Tracey, 2007), while the ventromedial region plays a causal role in the learning of fear as well as in the extinction of fear (Battaglia et al., 2020). One neuroimaging study on dysmenorrheic Caucasian females also reported that females with and without long-term menstrual pain differ in terms of the central processing of acute pain in the entorhinal cortex (part of the DMN) (Vincent et al., 2011).

In the current study, our aim was to investigate the brain responses evoked by heat pain to clarify the central mechanisms of pain processing among young Asian PDM females. We hypothesized that brain regions involved in pain processing/modulation and central sensitization may already have developed adaptive central mechanisms in response to cyclic menstrual pain. It is possible that central sensitization manifests as increased responsiveness to noxious stimuli in several pain-processing regions of the brain (Zambreanu et al., 2005). It has been suggested that central sensitization is maintained by the activity of the brainstem, such that any increase in the intensity of pain is reflected by activity in the primary somatosensory cortex (Lee et al., 2008). The DMN is generally not considered a part of the pain-processing network; however, it is integral to the dynamic pain connectome (Kucyi and Davis, 2015, 2017) and has been implicated in shaping individual differences in pain sensitivity (Emerson et al., 2014; Zhang X. et al., 2020). Our findings in previous work indicated that the DMN plays an adaptive role in the cognitive modulation of menstrual pain *via* interactions among the resting-state brain networks (Wu et al., 2016). Adaptive neuroplasticity in these brain regions may partly contribute to the absence of central sensitization and play a role in preserving the integrity of the functional brain architecture among young Asian PDM females (Lee et al., 2014, 2018).

## Materials and methods

### Subjects

The subjects in this study comprised a subset of the participants from our previous genetic/behavioral study of PDM (Lee et al., 2014), including those who were eligible for neuroimaging analysis of evoked brain responses to heat pain. The inclusion criteria were as follows: (1) 20–30-year-old Asian (Taiwanese) female; (2) a regular menstrual cycle of approximately 27–32 days; (3) a history of menstrual pain longer than 6 months; (4) average menstrual pain under regular treatment with a rating higher than four on a verbal numeric rating scale (NRS, 0 = not at all, 10 = the worst imaginable pain) in the last 6 months; and (5) right-handedness, as confirmed by the Edinburgh Handedness Inventory (Oldfield, 1971). The primary exception was the experience of menstrual pain intensity rated from none to mild (defined as NRS < 3). All PDM females were clinically examined and diagnosed in the gynecology clinic by the same certified gynecologist (H-TC) and underwent pelvic ultrasonography to exclude cases of secondary dysmenorrhea caused by organic pelvic diseases, such as endometriosis or adenomyosis. The inclusion criteria for healthy control females were similar to those for the PDM group, except that the subjects in the control group had no pain whatsoever during menses (NRS = 0). The exclusion criteria for all participants were as follows: (1) use of oral contraceptives, hormonal supplements, Chinese herbal medicine, or any centrally acting medication (e.g., opioid, anti-epileptics) within 6 months prior to the study; (2) pathological pituitary gland disease; (3) organic pelvic disease; (4) any psychiatric or neurological disorders (e.g., premenstrual dysphoric disorder); (5) previous brain surgery or head injury involving loss of consciousness; (6) immediate plans for pregnancy or a positive pregnancy test; (7) a history of childbirth; and (8) having a metal/pacemaker implant, claustrophobia, or any contraindications related to MRI. Note that no analgesics had been used by the subjects within 24 h prior to the study. The study was conducted in accordance with the Declaration of Helsinki under approval by the Institutional Review Board of Taipei Veterans General Hospital. All subjects signed a written informed consent form prior to participation in the study.

The initial enrollees included 106 PDM and 102 healthy control females who fulfilled the inclusion and exclusion criteria. Following enrollment in the neuroimaging experiments, 15 PDM and four control females were excluded due to incidental brain findings [e.g., normal brain variants, such as cavum septum pellucidum; and brain abnormalities, such as arachnoid cysts; for more details, see Li et al. (2015)], 9 PDM and two control females were excluded due to other physical conditions, 28 PDM and 39 control females were excluded because they did not complete neuroimaging experiments in both the menstrual and periovulatory phases, and 21 PDM and 21 control females were excluded for further neuroimaging analysis owing to significant head motion (translation >1.5 mm or rotation >1.5°) during the event-related fMRI scan. In addition, 2 PDM and four control females were excluded due to technical problems related to hormonal measurements. This resulted in the inclusion of 31 otherwise healthy females with PDM (age, 22.8 ± 2.67 years) and 32 education-matched, healthy control females (age, 23.4 ± 2.09 years) (see Table 1 for demographic data and Supplementary Figure 1 for the recruitment of subjects).

**TABLE 1:** Demographic data and baseline information of PDM and control groups.

	Control (*n* = 32)	PDM (*n* = 31)	*P-*value
Age (year)	23.4 ± 2.09	22.8 ± 2.67	0.098
Age at menarche (year)	12.4 ± 1.32	12.2 ± 1.35	0.801
Years of menstruation	11.0 ± 2.52	10.6 ± 3.09	0.498
Days of one menstrual cycle	29.5 ± 1.06	29.3 ± 1.37	0.750
**Menstrual pain experience**			
Years of dysmenorrhea history		8.6 ± 3.02	
Days of menstrual pain per cycle		2.2 ± 0.69	
Overall PRI (inception of study; range, 0–78)		34.4 ± 14.98	
Overall PPI (inception of study; range, 0–5)		3.0 ± 1.21	
Current PRI (MENS phase; range, 0–78)		30.0 ± 12.53	
Current PPI (MENS phase; range, 0–5)		2.7 ± 1.01	

PDM, primary dysmenorrhea; PPI, present pain intensity of the McGill Pain Questionnaire; PRI, pain rating index of the McGill Pain Questionnaire; MENS phase, menstrual phase. Data are presented as the means ± SD.

### Experiment design

All of the subjects in the two groups underwent blood sampling for serum gonadal hormone assays, quantitative sensory testing, and brain MRI scans (T1 and event-related fMRI scanning of noxious heat stimulation) during the menstrual phase (i.e., painful phase, days 1–3 of the menstrual cycle) and periovulatory phase (i.e., non-painful phase, days 12–16 of the menstrual cycle). Ovulation was confirmed using a urinary luteinizing hormone test (Han Chiun Proper LH Rapid Test) to verify that the subjects were scanned during their periovulatory period.

### Serum gonadal hormone assays

Sera extracted from blood samples drawn during the respective menstrual and periovulatory phases were stored for batch analysis using commercialized assays (UniCel DxC 800 Synchron Clinical Systems, Beckman Coulter, Inc., Brea, CA, United States). The total serum concentrations were determined using chemiluminescence immunoassays for estradiol and progesterone as well as radioimmunoassays for testosterone.

### Quantitative sensory testing

Pain sensitivity throughout the menstrual cycle was quantitatively investigated by assessing thermal detection and pain thresholds in accordance with the established protocol (Rolke et al., 2006). Briefly, heat and cold stimuli were administered using a thermal stimulator (TSA 2001-II, MEDOC, Ramat Yishai, Israel) to bilateral periumbilical areas (T11-dermatome, referral area of menstrual pain) and forearm extensor areas (C7-dermatome, remote control area) during the menstrual and periovulatory phases. In each measurement, the baseline temperature of the thermode was set at 32°C. From this baseline temperature, all thresholds were obtained using the ramped stimulation method (1°C/sec). An ascending limit was used for heat simulation with the safety limit temperature set at 50°C for warm detection and pain. A descending limit was used for cold stimulation with the safety limit temperature set at 0°C for cold detection and pain. The temperature of the thermode increased or decreased to the target temperature (i.e., for the detection or pain thresholds) and returned to the baseline immediately. After determining the detection thresholds for cold and warm first, we then determined the pain thresholds for cold and heat. Mean threshold temperatures were calculated by averaging three consecutive measurements.

### Determining the individual-defined temperature indicating moderate heat pain

A thermal stimulator (TSA 2001-II, MEDOC, Israel) was attached to the left inner forearm using Velcro straps. In the menstrual and periovulatory phases, ramped stimulation (0.5°C/sec ascending from 32°C) was used to determine the minimum temperature at which that participant would describe the sensation as moderate pain at the level of NRS = 6. When participants entered the MRI room to undergo brain scans, the temperature indicating moderate heat pain was retested to ensure that their perception of heat pain intensity was unaffected by the environment. Matching pain intensity (set at NRS = 6) allowed us to investigate possible alterations in the central processing of an identical pain percept across menstrual cycle phases in females with and without PDM (Kucyi et al., 2016).

### Heat stimulation under fMRI

In the fMRI scanning session for evoked brain responses, non-painful warm stimuli (WARM; set at 38°C) and moderate heat-pain stimuli (PAIN; set at the defined temperature of moderate heat pain, i.e., NRS = 6) were applied to the left inner forearm of each participant. We employed an event-related design with a stimulus (WARM or PAIN) presentation of 2.5 sec and an inter-stimulus interval of 30 sec (BASELINE; set at a temperature of 32°C). In each session, 9 WARM and 9 PAIN stimuli were presented in random order with intervening BASELINE periods (see Supplementary Figure 2 for paradigm of experimental heat stimulation). Each experiment comprised two fMRI sessions, each of which used the same order of stimuli presentation. Each session took roughly 10 min. After completing each session, the participants were asked to rate the average pain intensity and describe the characteristics of the heat pain.

### Brain MRI scanning

Event-related fMRI images were acquired using a 3.0 Tesla MRI scanner (Magnetom Trio Tim, Siemens, Erlangen, Germany) *via* echo-planar imaging (EPI) with the following scanning parameters: repetition time (TR) = 2,500 ms, echo time (TE) = 30 ms, 40 axial slices/image volume with slice thickness = 3.4 mm, and flip angle = 90°. Each session of EPI scanning consisted of 260 volumes. All subjects were scanned with their eyes open in a supine and relaxed position. T1-weighted 3-dimensional structural images were acquired using a magnetization-prepared rapid-acquired gradient echo sequence (MPRAGE) with the following scanning parameters: TR = 2,530 ms, TE = 3.03 ms, inversion time (TI) = 1,100 ms, flip angle = 7°, field of view = 224 × 256 mm^2^, matrix size = 224 × 256, number of slices = 192, and slice thickness = 1 mm. Head cushions and earplugs were respectively used to reduce interference from head motion and ambient noise.

### Image preprocessing

All EPI images were preprocessed using Statistical Parametric Mapping software (SPM12, Wellcome Trust Centre for Neuroimaging, University College London, London, United Kingdom^1^) in MATLAB (The MathWorks, Inc., Natick, MA, USA), as follows: correction of slice timing, realignment for head motion correction (6-parameter rigid body transformation), and spatial normalization. The time course of head motion for each subject was obtained by estimating the translation and rotation along each axis for 260 consecutive EPI volumes. Head motion can have a profound influence on fMRI analysis (Van Dijk et al., 2012); therefore, we excluded subjects who presented significant head motion (translation >1.5 mm or rotation >1.5°) of any volume from further analysis. Among the subjects that were retained for fMRI analysis (31 PDM and 32 control females), we found no main effects of group (PDM vs. control), menstrual cycle phase (menstrual phase vs. periovulatory phase), or interaction between them in terms of the root mean squares of overall translation and rotation parameters of head motion (all *P* > 0.05). The EPI images were spatially normalized using the SPM’s standard EPI template in the Montreal Neurological Institute (MNI) space and re-sampled to an isotropic voxel size of 2 × 2 × 2 mm^3^. The normalized images were then spatially smoothed using a 3D Gaussian kernel with a full width at half-maximum (FWHM) of 8 mm.

### First-level fMRI analysis

First-level parameter estimates were compared using linear contrast (*t*-contrast) with temporal derivatives. Timing parameters were set as follows: inter-scan interval = 2.5 sec and microtime resolution = 40 with onset = 21. Head motion parameters estimated from rigid-body realignment were added as regressors of no interest to reduce the influence of head motion on fMRI analysis (Van Dijk et al., 2012). The default high-pass temporal filter was applied in SPM (cut-off period: 128 sec; 0.0078 Hz) to attenuate noise. Note that noxious heat stimuli can elicit perceptions other than pain. To avoid potential confounding effects of administering heat (i.e., focus on pain-specific effects) (Duerden and Albanese, 2013; van den Bosch et al., 2013), we created a PAIN versus WARM contrast for use in second-level analysis.

### Second-level fMRI analysis: Group comparisons

The evoked brain responses related to PAIN versus WARM contrast were computed using one-sample *t*-tests for the PDM and control groups in the menstrual and periovulatory phases. We performed between-group comparisons to address the *state* (control vs. PDM in the menstrual phase) and *trait* (control vs. PDM in the periovulatory phase) effects of cyclic menstrual pain on evoked brain responses to noxious heat. *State*-related effects are acute menstrual pain-primed, whereas *trait*-related effects exist even without acute menstrual pain. Gonadal hormones may have an influence on pain-related brain activation (Veldhuijzen et al., 2013), and trivial non-significant differences were noted between the individual-defined temperature of cutaneous heat stimulation in the PDM and control groups (Table 2); therefore, serum gonadal hormone levels (estradiol, progesterone, and testosterone) and temperature indicating moderate heat pain (NRS = 6) were entered as covariates in the statistical model of SPM to obtain pain-specific functional correlates. Significance was set at the uncorrected voxel level of *P* < 0.005, followed by a family-wise error rate-corrected cluster level of *P* < 0.05. The anatomic regions of cluster maxima that show significant differences in the evoked brain responses were labeled according to the Talairach Daemon database (Lancaster et al., 2000) and Automated Anatomical Labeling (AAL) atlas (Tzourio-Mazoyer et al., 2002).

**TABLE 2:** Results of repeated-measures ANOVA of quantitative sensory testing and temperature of moderate heat pain: Effects of group and menstrual cycle phase.

			Main effect		Interaction
	Control (*n* = 32)	PDM (*n* = 31)	Group (*P*)	Phase (*P*)	Group*Phase (*P*)
**Heat pain threshold–C7 (°C)**					
MENS phase	45.0 ± 3.33	44.0 ± 3.55	0.329	0.868	0.606
POV phase	44.8 ± 3.51	44.2 ± 2.92			
**Heat pain threshold–T11 (°C)**					
MENS phase	43.9 ± 3.20	43.1 ± 3.15	0.243	0.101	0.554
POV phase	44.4 ± 3.08	43.4 ± 3.14			
**Cold pain threshold–C7 (°C)**					
MENS phase	9.1 ± 10.67	11.9 ± 10.52	0.458	0.887	0.355
POV phase	10.2 ± 10.73	11.1 ± 11.14			
**Cold pain threshold–T11 (°C)**					
MENS phase	11.0 ± 11.07	13.6 ± 11.45	0.681	0.872	0.097
POV phase	12.7 ± 10.98	12.2 ± 10.59			
**Temperature of moderate heat pain (°C)**					
MENS phase	45.2 ± 2.29	45.3 ± 1.98	0.700	0.072	0.643
POV phase	44.7 ± 2.88	45.0 ± 1.94			

ANOVA, analysis of variance; PDM, primary dysmenorrhea; MENS phase, menstrual phase; POV phase, periovulatory phase. Data are presented as the means ± SD.

### Correlation between evoked brain responses and the temperature associated with moderate heat pain

The temperature of noxious heat can influence the magnitude and extent of brain activation in healthy controls (Becerra et al., 1999; Kong et al., 2010); therefore, we examined the relationship between the evoked brain response of significant clusters and the temperature associated with moderate heat pain in the control and PDM groups. The evoked brain response of each cluster that showed a significant difference for the between-group comparisons of noxious heat stimulation was extracted. Two-tailed partial correlation analysis was performed with serum estradiol, progesterone, and testosterone levels entered as covariates. The significance level for the partial correlation analysis was set at *P* < 0.05.

### Statistical analysis

Two-sample *t*-tests or the Mann–Whitney *U* tests (if the data did not conform to normal distribution) were used to examine between-group differences in demographic data. In assessing serum gonadal hormone levels, quantitative sensory testing, and the individual-defined temperature of moderate heat pain during two menstrual cycle phases, general linear models with a repeated-measures design were used to examine the possible effects of group (PDM vs. control) and menstrual cycle phase (menstrual phase vs. periovulatory phase) as well as the interaction between them. SPSS Statistics 20.0 (SPSS Inc., Chicago, IL, United States) was used for all statistical analysis. The data are presented as mean ± SD, and the results were considered significant at *P* < 0.05 (two-tailed).

## Results

### Demographic data

No significant between-group differences were observed in terms of age, age at menarche, years of menstruation, or the average duration of a menstrual cycle. The PDM group had a long history of cyclic menstrual pain (8.6 ± 3.02 years), with pain during a single menstrual cycle lasting 1–3 days (2.2 ± 0.69 days). The overall menstrual pain experience, as assessed by the pain rating index (34.4 ± 14.98) and present pain intensity (3.0 ± 1.21) of McGill Pain Questionnaire, confirmed that the PDM group experienced long-term cyclic menstrual pain of moderate to severe degree (Table 1).

### Serum gonadal hormone assays

We observed a significant main effect of menstrual cycle phase, but no main effect of group or the interaction between group and menstrual cycle phase, on serum estradiol, progesterone, and testosterone levels. The serum estradiol, progesterone, and testosterone levels were significantly higher during the periovulatory phase than during the menstrual phase in both the PDM and control groups (Supplementary Table 1).

### Quantitative sensory testing

In line with one previous report (Rolke et al., 2006), we observed no right-left differences in any of the measured thresholds of heat or cold pain. We therefore averaged the bilateral values of the corresponding dermatomes to perform group comparisons. The measured thresholds of heat and cold pain in our study cohort were similar to the values obtained among Caucasians (Rolke et al., 2006). No main effects of menstrual cycle phase, group, or interactions between them were observed in the measured thresholds of heat or cold pain in the respective T11- and C7-dermatomes (Table 2). In accordance with our previous report with a larger sample (Lee et al., 2014), we detected no regional or generalized hypersensitivity to cutaneous thermal pain among the young Asian PDM females.

### Evoked brain responses to heat pain

We observed no main effects of menstrual cycle phase, group, or interaction between them in terms of the minimum temperature indicating moderate heat pain (i.e., the temperature that elicited heat pain of NRS = 6 during fMRI scanning) (Table 2). The stimulation paradigm of cutaneous heat pain revealed pain-related brain activation in the anterior and posterior cingulate gyrus, insula, postcentral and precentral gyri, cerebellum, thalamus, and basal ganglia associated with PAIN versus WARM contrast (at family-wise error rate-corrected cluster level of *P* < 0.05). Our findings were in line with a previous meta-analysis on the localization of heat pain-related brain activation (Duerden and Albanese, 2013). Average group responses to cutaneous heat pain in the menstrual and periovulatory phases are listed in Supplementary Tables 2–5 and Supplementary Figures 3–6.

**FIGURE 1:**
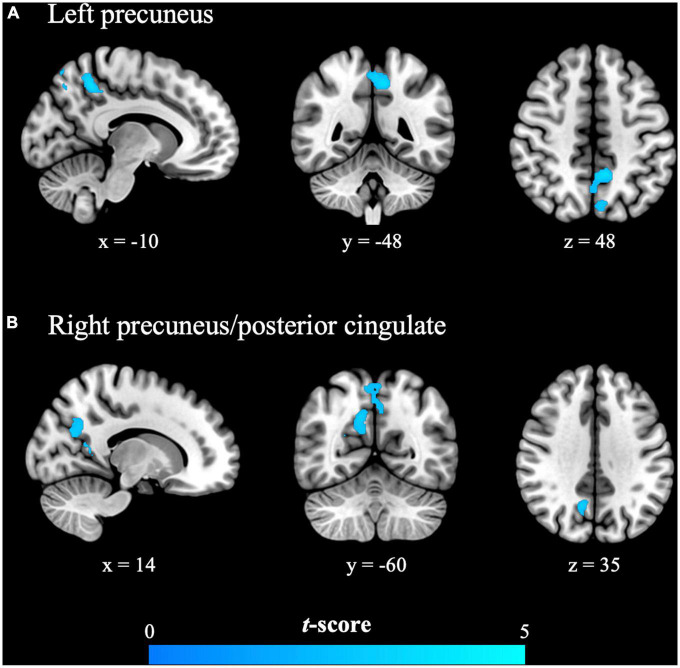
The primary dysmenorrhea group presented significantly reduced evoked brain responses to cutaneous heat pain in the **(A)** left precuneus and **(B)** right precuneus/posterior cingulate during the painful menstrual phase. Significance was set at the uncorrected voxel level of *P* < 0.005, followed by the family-wise error rate-corrected cluster level of *P* < 0.05. The cold color (blue) indicates the observed reduction in the evoked brain responses in females with primary dysmenorrhea.

In the menstrual phase (painful phase), brain responses to cutaneous heat pain in the PDM group in the left precuneus and right precuneus/posterior cingulate [i.e., posterior part of the DMN (pDMN)] were significantly lower than in the control group (Table 3 and Figure 1). In the periovulatory phase (non-painful phase), no significant differences were observed between the groups in terms of brain responses to cutaneous heat pain. PDM females experiencing acute menstrual pain presented a blunted pDMN response to experimental heat pain, which is a phenomenon that is unlikely to occur without concomitant menstrual pain.

**TABLE 3:** Significant reductions in evoked brain responses to cutaneous heat pain in the PDM group (menstrual phase).

Regions of reduced response	BA	Cluster size (voxels)	*t* score	*z* score	Peak MNI coordinate		
					*x*	*y*	*z*
Left precuneus	7/31	589	3.81	3.58	−10	−48	48
Right precuneus/posterior cingulate	31/7	255	3.79	3.56	12	−62	34

BA, Brodmann area; MNI, Montreal Neurological Institute; PDM, primary dysmenorrhea.

### Correlation between evoked brain responses and the temperature of moderate heat pain

**FIGURE 2:**
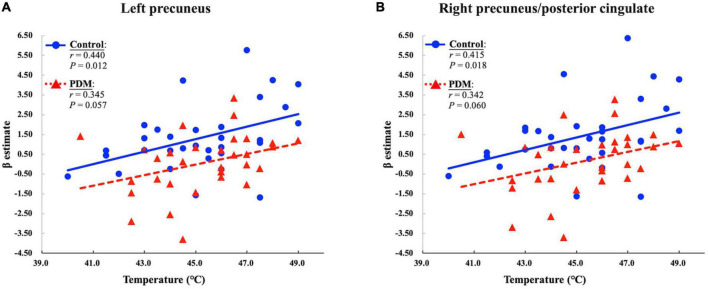
The primary dysmenorrhea (PDM) group presented a reduced positive correlation between the individual-defined temperature of moderate heat pain and the responses evoked in the **(A)** left precuneus and **(B)** right precuneus/posterior cingulate during the painful menstrual phase. *P* and *r* values for the partial correlation analysis in the control and PDM groups are presented.

Evoked brain responses in clusters presenting significant between-group differences during the menstrual phase were extracted to examine their correlation with the temperature indicating moderate heat pain (NRS = 6). In the control group, the evoked pDMN response during the menstrual phase was positively correlated with the temperature of moderate heat pain (*P* = 0.012 and *r* = 0.440 in the left precuneus; *P* = 0.018 and *r* = 0.415 in the right precuneus/posterior cingulate) after controlling for fluctuations in serum gonadal hormone levels. This positive correlation is in agreement with a previous fMRI study, which reported a correlation between the temperature deemed noxious and the degree of activation in the posterior cingulate gyrus (Becerra et al., 1999). Note, however, that this positive correlation was not observed in the PDM group (*P* = 0.057 and *r* = 0.345 in the left precuneus; *P* = 0.060 and *r* = 0.342 in the right precuneus/posterior cingulate) (Figure 2). PDM females experiencing acute menstrual pain presented a de-coupling between the minimum temperature that elicited moderate heat pain and the brain responses evoked in the pDMN.

### Correlation between evoked brain responses and PDM characteristics

We also investigated the influence of PDM characteristics on evoked brain responses to noxious heat by examining the correlation of the evoked pDMN responses that showed significant between-group differences with years of dysmenorrhea history as well as current menstrual pain experience in the PDM group. After controlling for fluctuations in serum gonadal hormone levels, we observed no significant correlation between evoked pDMN responses and dysmenorrhea history or pain rating index/present pain intensity of McGill Pain Questionnaire (all *P* > 0.05). The evoked responses to noxious heat in the pDMN were unrelated to the clinical characteristics of PDM, and the presence of acute menstrual pain *per se* is likely responsible for altered responses in the pDMN.

## Discussion

In the current study [an extension of our previous work; (Lee et al., 2014)], we found no firm evidence supporting the presence of central sensitization to noxious heat stimulation among young Asian PDM females in terms of quantitative sensory testing or neuroimaging data. The subjects did not exhibit heightened pain sensitivity to cutaneous thermal stimuli (quantitative sensory testing or temperature of moderate heat pain), during either the painful menstrual or non-painful periovulatory phases. Compared to healthy controls, the PDM subjects did not present elevated evoked responses (magnitude or spatial extent) in pain-processing brain regions or regions associated with central sensitization [e.g., brainstem and primary somatosensory cortex (Lee et al., 2008)] during the menstrual or periovulatory phases. Our findings are consistent with a recent study showing negative findings with respect to the sensitivity and neural processing of experimental visceral pain in PDM subjects (Böttcher et al., 2019).

During the menstrual phase, PDM females with acute menstrual pain exhibited significantly reduced responses to noxious heat in the pDMN. Note that in the menstrual phase, the positive correlation between evoked pDMN responses and the temperature of moderate heat pain in healthy controls was less pronounced in PDM females. These findings imply that young Asian PDM females experience a blunted response to the effects of noxious heat and a de-coupling of the pDMN during the painful menstrual phase (but not in the non-painful periovulatory phase). We speculate that this is an adaptive mechanism aimed at reducing the impact of cyclic menstrual pain on the brain, which may have an inhibitory effect on central sensitization. Brain regions of the pDMN are important mediators of pain in healthy subjects, which affect the relationships among stimuli intensity, pain rating/sensitivity, and evoked brain responses to noxious heat (Becerra et al., 1999; Atlas et al., 2014; Damascelli et al., 2021) or electric stimuli (Goffaux et al., 2014). Previous structural MRI studies also reported a correlation between changes in gray matter in the pDMN and sensitivity to noxious heat (Emerson et al., 2014; Zhang X. et al., 2020). This study provides further evidence that the pDMN is functionally engaged in the processing of noxious heat stimulation in healthy subjects (Becerra et al., 1999).

A secondary issue in the current study was resting-state neuronal activity in the clusters of pDMN presenting significantly reduced responses to noxious heat in PDM subjects (i.e., the left precuneus and right precuneus/posterior cingulate). This involved computing regional spontaneous neuronal activity using an amplitude of low-frequency fluctuation (ALFF) (Zang et al., 2007) and a fractional amplitude of low-frequency fluctuation (fALFF) (Zou et al., 2008) in conjunction with the local synchronization of resting-state brain activity using regional homogeneity (ReHo) (Zang et al., 2004). The average ALFF, fALFF, and ReHo values in the two pDMN clusters showed no significant between-group differences during the menstrual phase (Supplementary Table 6). These findings provide additional evidence that the altered responses to noxious heat cannot be ascribed to the altered resting activity in the pDMN. It further supports the assertion that pDMN plays an adaptive rather than maladaptive role in response to cyclic menstrual pain among PDM females.

Several studies have reported that PDM females exhibit functional and structural alterations in brain regions of the DMN. PDM females presented alterations in metabolism, spontaneous neuronal activity, cerebral blood flow, and functional connectivity in the precuneus and posterior cingulate cortex (Tu et al., 2009; Jin et al., 2017; Liu et al., 2017; Zhang et al., 2019; Zhang Y. N. et al., 2020). PDM females also presented alterations in gray matter volume and cortical thickness in the precuneus. Note that the extent of the alterations is correlated with the duration and menstrual pain experience of PDM (Tu et al., 2010, 2013; Liu et al., 2016). More importantly, previous studies on cross-network interactions have reported that the DMN exhibits adaptive responses to cyclic menstrual pain in PDM females (Wu et al., 2016; Dun et al., 2017). Reduced functional coupling of the DMN from the salience network implies that the interoceptive awareness of pain and attention to pain are inhibited (Wu et al., 2016; Dun et al., 2017). Furthermore, enhanced functional coupling between the DMN and the executive control network is an indication of adaptive functional reorganization enhancing the cognitive modulation of menstrual pain among these young Asian PDM females (Wu et al., 2016). In light of evoked brain responses to acute pain and resting-state cross-network interactions, this study provides important evidence supporting the assertion that the DMN plays an adaptive role in the clinical manifestations observed in young Asian PDM females. We speculate that the adaptive neuroplasticity of the DMN in response to cyclic menstrual pain in this and previous studies (Wu et al., 2016; Dun et al., 2017) helps to maintain the overall integrity of the resting-state functional brain architecture, leading to normal psychosocial outcomes (Lee et al., 2018).

It appears that ethnic and genetic characteristics may play roles in the clinical manifestations (e.g., pain sensitivity) observed among PDM females. Imaging genetics studies of functional connectivity have indicated that the intricate interactions between the descending pain modulatory systems and genetic polymorphisms, including *BDNF* Val66Met (Wei et al., 2016b) and *OPRM1* A118G (Wei et al., 2017), may underpin individual differences in susceptibility to pain among Asian PDM females. Note that descending pain modulatory systems (periaqueductal gray matter) and DMN (posterior cingulate cortex) may be functionally linked in the endogenous modulation of pain (Zyloney et al., 2010) and associated negative psychological effects (e.g., pain rumination) (Kucyi et al., 2014). In the context of genetics, we speculate that the adaptive responses of the pDMN in PDM females during the menstrual phase may influence descending pain modulation to inhibit pain hypersensitivity. In the future, it may be possible to use imaging genetics in combination with noxious stimulation to delineate the mechanisms underlying ethnic or individual differences in the experience of pain and to clarify the mechanisms underlying the presence or absence of central sensitization among PDM females.

Our findings on dysmenorrheic Asian females differ from those in a previous fMRI study on dysmenorrheic Caucasian females (Vincent et al., 2011). In that study, the dysmenorrheic subjects exhibited enhanced evoked responses to noxious heat stimulation in the left entorhinal cortex (part of the DMN) during non-menstrual phases, and the effects were positively correlated with the severity of acute menstrual pain. In terms of ethnic differences, it appears that differences in the central processing of noxious heat play a role in the contradictory manifestations of pain sensitivity (i.e., absence or presence of central sensitization) between Asian and Caucasian dysmenorrheic females. Note that the discrepancies between the previous findings and those in the current study may be attributed to differences in the analytic methods and demographic characteristics of recruited subjects. In the current study, we limited the study cohort to PDM subjects with a confirmed diagnosis by a gynecologist, whereas the previous study may have included subjects with secondary dysmenorrhea. The mean age of subjects in the current study (23–24 years) was also younger than in the previous study (30–32 years of age). In the current study, pain-specific effects were examined by analyzing evoked brain responses derived from the PAIN versus WARM contrast, whereas Vincent et al. (2011) investigated the evoked brain responses to noxious heat directly (i.e., without eliminating the effects of temperature). Nonetheless, both studies indicated the presence of altered DMN responses to pain among young women with long-term dysmenorrhea. It may be necessary to perform longitudinal follow-up studies to track the functional and structural alterations of DMN in response to cyclic menstrual pain.

Several limitations and future direction should be considered in the interpretation of our findings. To begin with, we used only noxious heat stimulation to investigate the effects of cyclic menstrual pain on pain responses in the brain. Future studies should use other methods of pain provocation to verify the generalizability of the current findings. It has been suggested that cold stimuli can predispose the subject to menstrual pain (Wang et al., 2022); however, the psychophysical findings of cold stimulation-based experiments have been inconsistent, particularly among studies of central sensitization in dysmenorrhea (Slater et al., 2015; Payne et al., 2019). Second, the subjective experience of pain and sensitivity to temperature can vary considerably among individuals, and researchers have reported high inter-subject variability in brain responses evoked by noxious stimuli (Davis et al., 1998). Genetic, psychological, sensory-cognitive, neuroanatomical, and environmental (including cultural and ethnic) factors may all contribute to the individual differences in the experience of pain (Apkarian et al., 2005; Kupers and Kehlet, 2006; Norbury et al., 2007; Erpelding et al., 2012; Emerson et al., 2014; Zhang X. et al., 2020) and variations in the clinical manifestations of pain among different PDM populations (Tu et al., 2009, 2010, 2013; Wei et al., 2016a,b, 2017; Wu et al., 2016). In the current experimental study, we did not address the complex interdependence of these factors. These issues will have to be taken into consideration in future research.

## Conclusion

This study observed a blunted evoked response to noxious heat and a de-coupling of the pDMN among PDM females experiencing acute menstrual pain. These results indicate the presence of an adaptive mechanism reducing the impact of cyclic menstrual pain on the brain. This mechanism may also have contributed to the absence of central sensitization observed among these young Asian PDM females. Variations in clinical manifestations among different PDM populations can be attributed to distinct central processing and pain modulation in response to cyclic menstrual pain. The findings in this preliminary report will have to be verified in neuroimaging studies of PDM using a large sample of subjects with different ethnic attributions and a wider range of sensory modalities for pain provocation.

## Data availability statement

The raw data supporting the conclusions of this article will be made available by the authors, without undue reservation.

## Ethics statement

The studies involving human participants were reviewed and approved by the Institutional Review Board of Taipei Veterans General Hospital. The patients/participants provided their written informed consent to participate in this study.

## Author contributions

L-CL: conceptualization, investigation, formal analysis, data curation, writing—original draft, and visualization. Y-YC: conceptualization, investigation, formal analysis, data curation, and writing—original draft. W-CL and IL: investigation and data curation. C-JY: investigation and formal analysis. C-HL: formal analysis. H-TC: investigation and resources. L-FC: conceptualization, methodology, resources, and funding acquisition. J-CH: conceptualization, methodology, resources, writing—review and editing, supervision, project administration, and funding acquisition. All authors contributed to the article and approved the submitted version.

## Footnotes

1
http://www.fil.ion.ucl.ac.uk/spm
