# Supplementary material for: Adaptive neuroplasticity in the default mode network contributing to absence of central sensitization in primary dysmenorrhea
Source: Front Neurosci. 2023 Feb 9;17:1094988. doi: 10.3389/fnins.2023.1094988 (PMC9947468; doi:10.3389/fnins.2023.1094988)
Supplement: Supplementary file 1 [file Data_Sheet_1.DOCX]

**Supplementary Material**

**Figure S1. Recruitment of subjects for PDM and control groups
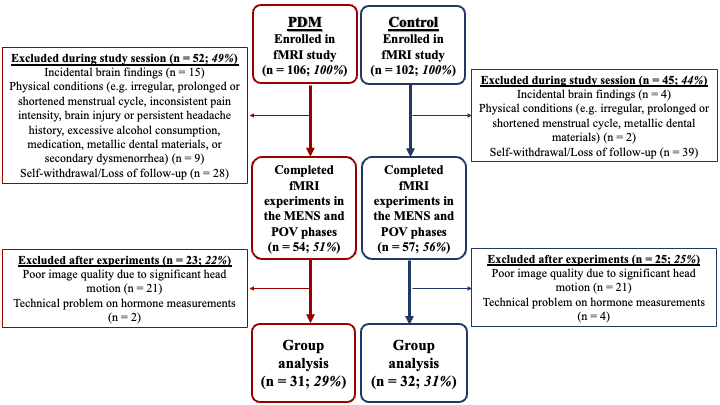
**

**Abbreviations**: fMRI, functional magnetic resonance imaging; PDM, primary dysmenorrhea; MENS phase, menstrual phase; POV phase, periovulatory phase.

**Figure S2. Experiment paradigm involving heat stimulation during fMRI**
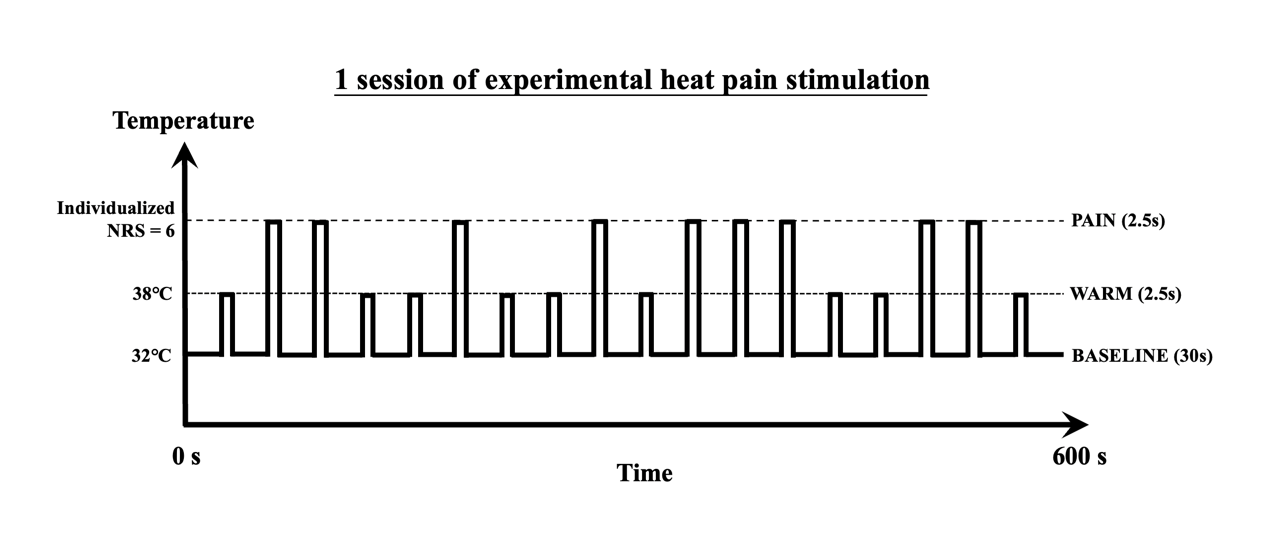


Non-painful warm stimuli (WARM; 38˚C for 2.5 secs) and individual-defined temperature of moderate heat-pain stimuli (PAIN; temperature at numeric rating scale [NRS] = 6 for 2.5 secs) were applied to the left inner forearm, with an inter-stimulus interval of 30 secs (BASELINE; 32˚C). In each session, nine WARM and nine PAIN stimuli were presented in random order with intervening BASELINE periods. The experiment included two functional sessions, in which the same order of stimuli presentation was followed. fMRI, functional magnetic resonance imaging.

**Figure S3.** Significant brain activation in response to cutaneous heat pain (under the PAIN versus WARM contrast) in the control group during the menstrual phase.
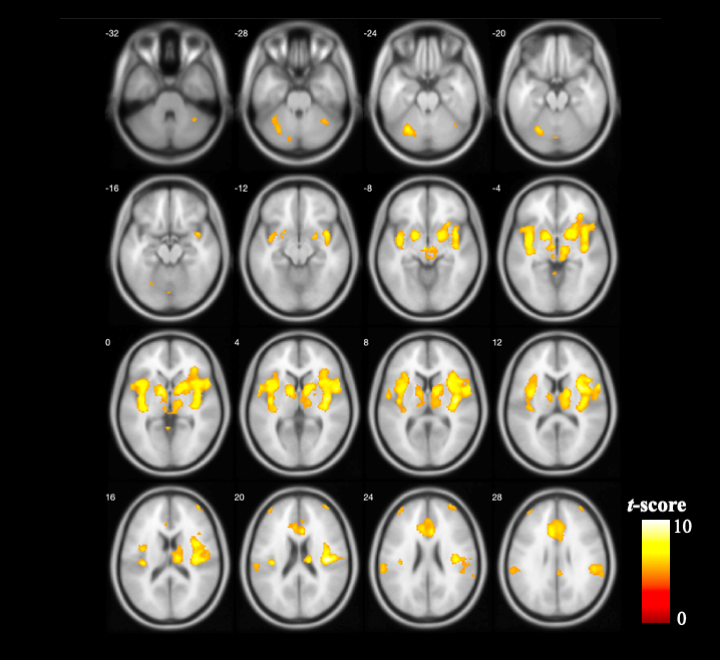


Significance was set at a family-wise error rate-corrected cluster level of *P* < 0.05, with a cluster size of > 10 voxels.

PAIN condition, brain activations at the individual-defined temperature of moderate heat pain (numeric rating scale = 6); WARM condition, brain activations at a temperature of 38˚C.

**Figure S4.** Significant brain activation in response to cutaneous heat pain (under the PAIN versus WARM contrast) in the control group during the periovulatory phase.
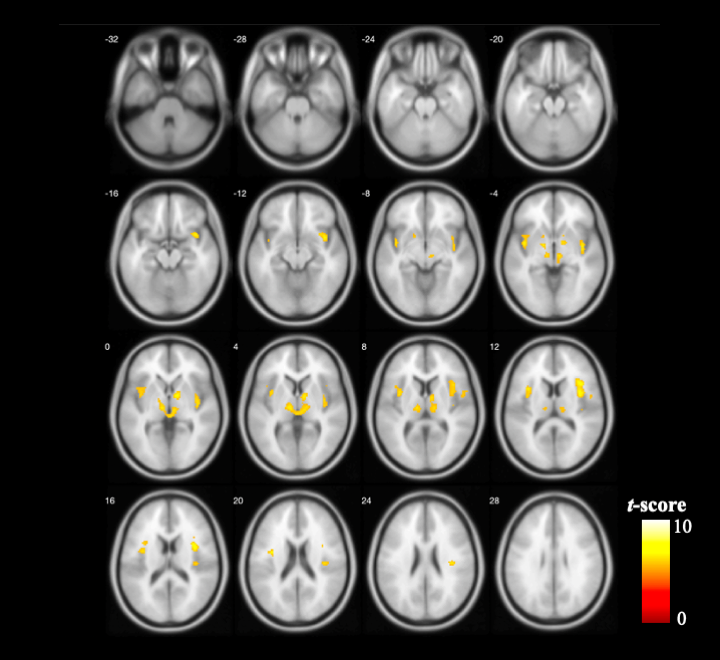


Significance was set at a family-wise error rate-corrected cluster level of *P* < 0.05, with a cluster size of > 10 voxels.

PAIN condition, brain activations at the individual-defined temperature of moderate heat pain (numeric rating scale = 6); WARM condition, brain activations at a temperature of 38˚C.

**Figure S5.** Significant brain activation in response to cutaneous heat pain (under the PAIN versus WARM contrast) in the primary dysmenorrhea group during the menstrual phase.
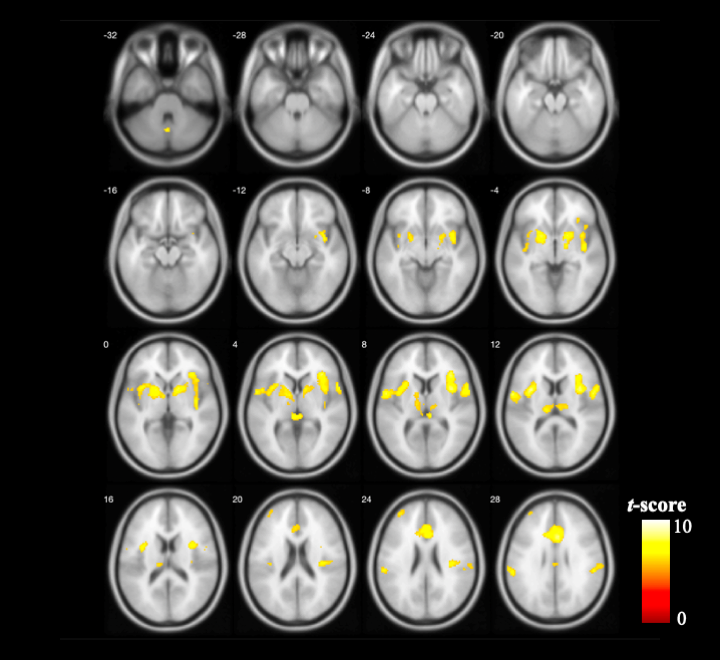


Significance was set at a family-wise error rate-corrected cluster level of *P* < 0.05, with a cluster size of > 10 voxels.

PAIN condition, brain activations at the individual-defined temperature of moderate heat pain (numeric rating scale = 6); WARM condition, brain activations at a temperature of 38˚C.

**Figure S6.** Significant brain activation in response to cutaneous heat pain (under the PAIN versus WARM contrast) in the primary dysmenorrhea group during the periovulatory phase.
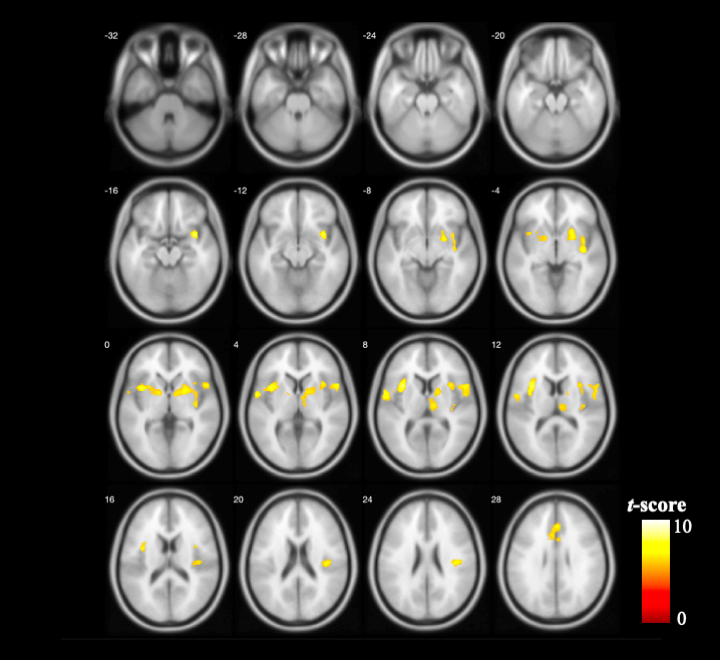


Significance was set at a family-wise error rate-corrected cluster level of *P* < 0.05, with a cluster size of > 10 voxels.

PAIN condition, brain activations at the individual-defined temperature of moderate heat pain (numeric rating scale = 6); WARM condition, brain activations at a temperature of 38˚C.

| **Table S1.** Repeated-measures ANOVA results of gonadal hormone levels: Effects of group and menstrual cycle phase | | | | | | | | |
| --- | --- | --- | --- | --- | --- | --- | --- | --- |
|  |  | |  |  | | **Main Effect** | | **Interaction** |
|  |  | | **Control** (n = 32) | | **PDM** (n = 31) | **Group** (*P*) | **Phase** (*P*) | **Group*Phase** (*P*) |
| **Estradiol** (pg/mL) | | |  | |  |  |  |  |
|  | | MENS phase | 41.5 ± 21.67 | | 32.2 ± 14.08 | 0.973 | < 0.001 | 0.453 |
|  | | POV phase | 119.6 ± 116.22 | | 129.7 ± 83.90 |  |  |  |
| **Progesterone** (ng/mL) | | |  | |  |  |  |  |
|  | | MENS phase | 0.4 ± 0.23 | | 0.5 ± 0.43 | 0.640 | 0.020 | 0.316 |
|  | | POV phase | 0.8 ± 1.18 | | 0.7 ± 0.50 |  |  |  |
| **Testosterone** (ng/mL) | | |  | |  |  |  |  |
|  | | MENS phase | 0.4 ± 0.19 | | 0.4 ± 0.21 | 0.180 | < 0.001 | 0.283 |
|  | | POV phase | 0.5 ± 0.16 | | 0.5 ± 0.28 |  |  |  |
| **Abbreviations**: ANOVA, analysis of variance; PDM, primary dysmenorrhea; MENS phase, menstrual phase; POV phase, periovulatory phase. Data are presented as the means ± SD. | | | | | | | | |

| **Table S2.** Significant brain activation in response to cutaneous heat pain (under the PAIN versus WARM contrast) in the control group during the menstrual phase | | | | | | | |
| --- | --- | --- | --- | --- | --- | --- | --- |
| **Cluster-level *P*** | **Cluster size** | ***t* score** | ***z* score** |  | **Peak MNI coordinate** |  | **Anatomical labeling** |
| **FWE-corrected** | **(voxels)** |  |  | **x** | **y** | **z** |  |
| < 0.001 | 6,636 | 11.38 | 7.09 | 40 | -18 | 20 | Right insula |
| < 0.001 | 2,073 | 9.11 | 6.31 | -40 | -12 | 2 | Left insula |
| < 0.001 | 2,636 | 8.66 | 6.13 | -6 | 18 | 30 | Left anterior cingulate cortex |
| < 0.001 | 303 | 7.69 | 5.71 | -26 | -66 | -24 | Left cerebellum |
| < 0.001 | 66 | 7.34 | 5.55 | -28 | -66 | -50 | Left cerebellum |
| < 0.001 | 99 | 7.02 | 5.39 | 40 | 56 | 18 | Right superior frontal gyrus |
| < 0.001 | 166 | 6.85 | 5.31 | -62 | -28 | 24 | Left inferior parietal lobule |
| < 0.001 | 73 | 6.72 | 5.24 | 4 | -34 | 50 | Right paracentral lobule |
| 0.002 | 26 | 6.56 | 5.15 | -2 | -50 | -2 | Left cerebellum |
| < 0.001 | 62 | 6.51 | 5.13 | 36 | -54 | -30 | Right cerebellum |
| < 0.001 | 66 | 6.42 | 5.08 | -40 | 50 | 22 | Left middle frontal gyrus |
| 0.001 | 33 | 6.37 | 5.06 | 20 | -42 | 70 | Right postcentral gyrus |
| 0.002 | 27 | 6.22 | 4.98 | 4 | -36 | 30 | Right posterior cingulate cortex |
| 0.005 | 18 | 6.14 | 4.93 | 46 | -54 | 46 | Right inferior parietal lobule |
| 0.002 | 28 | 6.09 | 4.90 | 16 | -66 | 38 | Right precuneus |
| 0.007 | 14 | 6.09 | 4.90 | -2 | -78 | -18 | Left cerebellum |
| 0.010 | 10 | 5.93 | 4.81 | -16 | -36 | 42 | Left middle cingulate cortex |
| Significance was set at a family-wise error (FWE) rate-corrected cluster level of *P* < 0.05, with a cluster size of > 10 voxels.  **Abbreviations**: MNI, Montreal Neurological Institute; PAIN condition, brain activations at the individual-defined temperature of moderate heat pain (numeric rating scale = 6); WARM condition, brain activations at a temperature of 38˚C. | | | | | | | |

| **Table S3.** Significant brain activation in response to cutaneous heat pain (under the PAIN versus WARM contrast) in the control group during the periovulatory phase | | | | | | | | | | |
| --- | --- | --- | --- | --- | --- | --- | --- | --- | --- | --- |
| **Cluster-level *P*** | **Cluster size** | ***t* score** | ***z* score** |  | **Peak MNI coordinate** | | |  | **Anatomical labeling** | |
| **FWE-corrected** | **(voxels)** |  |  | **x** | | **y** | **z** | | |  |
| < 0.001 | 574 | 7.25 | 5.51 | 38 | | 4 | 14 | | | Right insula |
| < 0.001 | 356 | 6.55 | 5.15 | -34 | | 10 | 12 | | | Left insula |
| < 0.001 | 644 | 6.46 | 5.11 | -12 | | -16 | 4 | | | Left thalamus |
| < 0.001 | 249 | 6.21 | 4.97 | 4 | | -8 | 42 | | | Right middle cingulate cortex |
| 0.012 | 14 | 5.88 | 4.78 | -14 | | 8 | -6 | | | Left lentiform nucleus |
| 0.005 | 30 | 5.60 | 4.62 | 60 | | 6 | 8 | | | Right precentral gyrus |
| Significance was set at a family-wise error (FWE) rate-corrected cluster level of *P* < 0.05, with a cluster size of > 10 voxels.  **Abbreviations**: MNI, Montreal Neurological Institute; PAIN condition, brain activations at the individual-defined temperature of moderate heat pain (numeric rating scale = 6); WARM condition, brain activations at a temperature of 38˚C. | | | | | | | | | | |

| **Table S4.** Significant brain activation in response to cutaneous heat pain (under the PAIN versus WARM contrast) in the primary dysmenorrhea group during the menstrual phase | | | | | | | |
| --- | --- | --- | --- | --- | --- | --- | --- |
| **Cluster-level *P*** | **Cluster size** | ***t* score** | ***z* score** |  | **Peak MNI coordinate** |  | **Anatomical labeling** |
| **FWE-corrected** | **(voxels)** |  |  | **x** | **y** | **z** |  |
| < 0.001 | 1,476 | 8.82 | 6.15 | 36 | 6 | 10 | Right insula |
| < 0.001 | 1,590 | 8.73 | 6.11 | -34 | 6 | 12 | Left insula |
| < 0.001 | 2,617 | 8.60 | 6.06 | 6 | 14 | 30 | Right middle cingulate cortex |
| < 0.001 | 269 | 8.11 | 5.85 | 54 | 0 | 12 | Right precentral gyrus |
| < 0.001 | 124 | 7.60 | 5.63 | 36 | -22 | 22 | Right insula |
| < 0.001 | 125 | 7.30 | 5.49 | -60 | -34 | 30 | Left inferior parietal lobule |
| 0.001 | 38 | 6.91 | 5.30 | -2 | -64 | -36 | Left cerebellum |
| < 0.001 | 142 | 6.79 | 5.24 | 54 | -32 | 30 | Right inferior parietal lobule |
| < 0.001 | 70 | 6.66 | 5.18 | -2 | -22 | 32 | Left middle cingulate cortex |
| < 0.001 | 82 | 6.62 | 5.16 | -36 | 48 | 24 | Left middle frontal gyrus |
| 0.002 | 23 | 6.26 | 4.97 | -20 | -62 | -50 | Left cerebellum |
| 0.006 | 13 | 6.14 | 4.90 | -36 | -22 | 22 | Left insula |
| 0.006 | 13 | 6.02 | 4.84 | -32 | -54 | -48 | Left cerebellum |
| Significance was set at a family-wise error (FWE) rate-corrected cluster level of *P* < 0.05, with a cluster size of > 10 voxels.  **Abbreviations**: MNI, Montreal Neurological Institute; PAIN condition, brain activations at the individual-defined temperature of moderate heat pain (numeric rating scale = 6); WARM condition, brain activations at a temperature of 38˚C. | | | | | | | |

| **Table S5.** Significant brain activation in response to cutaneous heat pain (under the PAIN versus WARM contrast) in the primary dysmenorrhea group during the periovulatory phase | | | | | | | | | | |
| --- | --- | --- | --- | --- | --- | --- | --- | --- | --- | --- |
| **Cluster-level *P*** | **Cluster size** | ***t* score** | ***z* score** |  | **Peak MNI coordinate** | | |  | | **Anatomical labeling** |
| **FWE-corrected** | **(voxels)** |  |  | **x** | | **y** | **z** | |  | |
| < 0.001 | 513 | 8.74 | 6.12 | -34 | | 12 | 10 | | Left insula | |
| < 0.001 | 989 | 7.80 | 5.72 | 38 | | -16 | -4 | | Right insula | |
| < 0.001 | 141 | 7.70 | 5.68 | -58 | | 0 | 8 | | Left precentral gyrus | |
| < 0.001 | 1,094 | 7.43 | 5.55 | -4 | | 10 | 38 | | Left middle cingulate cortex | |
| < 0.001 | 180 | 7.18 | 5.44 | 42 | | -20 | 22 | | Right insula | |
| 0.002 | 28 | 6.73 | 5.21 | -28 | | -64 | -48 | | Left cerebellum | |
| < 0.001 | 275 | 6.61 | 5.15 | 56 | | 0 | 10 | | Right precentral gyrus | |
| 0.001 | 35 | 6.43 | 5.06 | 54 | | -34 | 32 | | Right inferior parietal lobule | |
| Significance was set at a family-wise error (FWE) rate-corrected cluster level of *P* < 0.05, with a cluster size of > 10 voxels.  **Abbreviations**: MNI, Montreal Neurological Institute; PAIN condition, brain activations at the individual-defined temperature of moderate heat pain (numeric rating scale = 6); WARM condition, brain activations at a temperature of 38˚C. | | | | | | | | | | |

| **Table S6**. Resting-state brain activity in regions showing significantly reduced responses to cutaneous heat pain in the PDM group (menstrual phase) | | | | |
| --- | --- | --- | --- | --- |
|  | | **Control** (n = 32) | **PDM** (n = 31) | ***P* value** |
| **Left precuneus** | |  |  |  |
|  | ALFF | 1.25 ± 0.420 | 1.08 ± 0.340 | 0.077 |
|  | fALFF | 1.18 ± 0.282 | 1.05 ± 0.342 | 0.114 |
|  | ReHo | 0.80 ± 0.219 | 0.75 ± 0.196 | 0.352 |
| **Right precuneus & posterior cingulate** | |  |  |  |
|  | ALFF | 0.65 ± 0.291 | 0.54 ± 0.283 | 0.153 |
|  | fALFF | 0.90 ± 0.381 | 0.92 ± 0.434 | 0.826 |
|  | ReHo | 0.72 ± 0.292 | 0.72 ± 0.254 | 0.929 |
| Two-sample *t*-tests or Mann-Whitney U tests (if the data did not conform to normal distribution) were conducted to identify between-group differences. Data are presented as the means ± SD.  **Abbreviations**: ALFF, amplitude of low-frequency fluctuation; fALFF, fractional amplitude of low-frequency fluctuation; ReHo, regional homogeneity; PDM, primary dysmenorrhea. | | | | |
